# Near-roadway air pollution, immune cells and adipokines among obese young adults

**DOI:** 10.1186/s12940-022-00842-2

**Published:** 2022-03-19

**Authors:** Md Mostafijur Rahman, Fei Fei Liu, Sandrah P. Eckel, Ishwarya Sankaranarayanan, Pedram Shafiei-Jahani, Emily Howard, Lilit Baronikian, Fred Sattler, Frederick W. Lurmann, Hooman Allayee, Omid Akbari, Rob McConnell

**Affiliations:** 1 Department of Preventive Medicine, Keck School of Medicine, University of Southern California, 2001 N. Soto Street Building: SSB, Los Angeles, CA 90032 USA; 2 Department of Molecular and Cellular Immunology, University of Southern California, Los Angeles, California USA; 3 Department of Medicine, Keck School of Medicine, University of Southern California, Keck School of Medicine, Los Angeles, CA USA; 4 Sonoma Technology, Inc., Petaluma, CA USA

**Keywords:** Near-roadway air pollution, Adipose tissue, Adipokines, T cells, Macrophage polarization

## Abstract

**Background:**

Air pollution has been associated with metabolic disease and obesity. Adipokines are potential mediators of these effects, but studies of air pollution-adipokine relationships are inconclusive. Macrophage and T cells in adipose tissue (AT) and blood modulate inflammation; however, the role of immune cells in air pollution-induced dysregulation of adipokines has not been studied. We examined the association between air pollution exposure and circulating and AT adipokine concentrations, and whether these relationships were modified by macrophage and T cell numbers in the blood and AT.

**Methods:**

Fasting blood and abdominal subcutaneous AT biopsies were collected from 30 overweight/obese 18–26 year-old volunteers. Flow cytometry was used to quantify T effector (Teff, inflammatory) and regulatory (Treg, anti-inflammatory) lymphocytes and M1 [inflammatory] and M2 [anti-inflammatory]) macrophage cell number. Serum and AT leptin and adiponectin were measured using enzyme-linked immunosorbent assay (ELISA). Exposure to near-roadway air pollution (NRAP) from freeway and non-freeway vehicular sources and to regional particulate matter, nitrogen dioxide and ozone were estimated for the year prior to biopsy, based on participants’ residential addresses. Linear regression models were used to examine the association between air pollution exposures and adipokines and to evaluate effect modification by immune cell counts.

**Results:**

An interquartile increase in non-freeway NRAP exposure during 1 year prior to biopsy was associated with higher leptin levels in both serum [31.7% (95% CI: 10.4, 52.9%)] and AT [19.4% (2.2, 36.6%)]. Non-freeway NRAP exposure effect estimates were greater among participants with greater than median Teff/Treg ratio and M1/M2 ratio in blood, and with greater M1 counts in AT. No adipokine associations with regional air pollutants were found.

**Discussion:**

Our results suggest that NRAP may increase serum leptin levels in obese young adults, and this association may be promoted in a pro-inflammatory immune cell environment in blood and AT.

**Supplementary Information:**

The online version contains supplementary material available at 10.1186/s12940-022-00842-2.

## Introduction

Childhood obesity is a major public health problem in the United States and is a strong risk factor for the development of metabolic diseases, such as type 2 diabetes, non-alcoholic fatty liver disease, and cardiovascular disease, which can lead to increased morbidity and mortality in adult life [1–5]. However, obesity by itself does not necessarily lead to disease. Anatomic distribution of adipose tissue (AT), levels of physical activity, and the inflammatory profile of AT play key roles in systemic metabolic disease [6, 7]. Adiponectin and leptin are two well-studied adipokines secreted by AT that regulate local tissue inflammation [8] and are associated with obesity-induced cardiometabolic diseases [9–12]. Leptin has been shown to upregulate secretion of multiple inflammatory cytokines, such as TNF-α, IL-6, and IL-12, and affects adaptive and innate immunity [13], whereas adiponectin has anti-inflammatory functions [14].

A growing number of epidemiological studies suggest that air pollutants, especially near-roadway air pollutants (NRAP), and other environmental exposures may act as obesogens in children [15–19]. Epidemiological studies have further identified ambient air pollution as a risk factor for impaired glucose metabolism and the development of diabetes [20–22]. Although the underlying mechanisms for these associations are still not clear, evidence from human and animal studies suggests that elevated levels of ambient air pollution can alter the metabolic profile of AT [23–25]. Furthermore, some epidemiological studies have reported associations between air pollution exposure and changes in circulating adipokines in humans, but these results have not been consistently observed [22, 26–33]. Although AT is an important source of pro-inflammatory adipokines, to date studies of air pollution and adipokines have been limited to measurements of their concentrations in blood. There has been little study in humans of the relationship of adipokines in AT with ambient air pollution exposure.

AT homeostasis is influenced by resident T-lymphocytes and macrophages, which can play important roles in modulating AT inflammation. AT homeostasis requires a balance of both these pro-inflammatory (M1) and anti-inflammatory (M2) macrophage subtypes. Excess M1 macrophage infiltration (M1 polarization) results in increased AT inflammation due to excessive production of pro-inflammatory adipocytokines and is associated with metabolic and vascular disease [34–37] . Emerging evidence indicates that increases in activated T cell populations in AT may contribute to obesity-associated metabolic disease by dysregulating systemic adipokines [38–40]. However, the role of AT T cells and macrophages in air pollution-induced metabolic dysregulation has not yet been studied.

We evaluated the effect of air pollution on AT homeostasis as assessed by circulating and tissue levels of the adipokines leptin and adiponectin. We also examined a novel hypothesis that inflammatory (M1 and T effector) and anti-inflammatory (M2 and T regulatory) cells in AT modify (positively and negatively, respectively) the effects of air pollution on adipokines.

## Material and methods

### Study design and population

This cross-sectional study included 30 overweight and obese 18–26 years old participants from a convenience sample of volunteers recruited primarily through Craigslist advertisements. Participants came from across Southern California including Los Angeles, Orange, Ventura, Kern, and Riverside counties. Participants were excluded if they had taken medications known to affect insulin and/or glucose metabolism or body composition, been diagnosed with any major chronic illness since birth, or had type 1 or type 2 diabetes. Demographic characteristics collected included sex, race and ethnicity. Smoking history was categorized as never or ever even a single cigarette. BMI was calculated using participant’s weight and height [BMI = Weight (lb)/Height^2^ (in)].

Written informed consent and assent were obtained from all participants. This study was approved by the Institutional Review Board of the University of Southern California.

### Sample collection: blood and subcutaneous adipose tissue

Blood and Subcutaneous AT samples were collected between March 2015 and July 2017. Participants were instructed to fast after 10 PM and were admitted the following morning to the inpatient USC Clinical Trials Unit (CTU). After an overnight fast, 10 mL of blood were collected in a purple top (EDTA anticoagulant) tube for flow cytometry; 10 mL of blood was collected in a red top tube, centrifuged, and a 1 ml aliquot was frozen at − 80 °C for subsequent measurement of adipokines in serum. Biopsies were performed as previously described [41]. Briefly, the abdominal skin was prepared with three betadine scrubs at the biopsy site in the right anterior axillary line at the level of the umbilicus. After infiltration of the dermis and superficial subcutaneous tissue with 1% lidocaine, a 6–7 mm incision was made in the skin with #11 Bard Parker scalpel. A 6-mm Bergström side-cutting needle (Micrins Surgical, Lake Forest, IL, USA) was introduced approximately 1–1.5 in. through the incision into the subcutaneous abdominal AT. Suction was then applied from a 60-cc syringe attached by irrigation tubing to the Bergström needle. Four cuts were made with the cutting trochar as the needle was further advanced and rotated 90 degrees prior to each cut. The procedure consistently yielded 1.5-8 g of AT. Manual compression was applied to the wound and the incision was closed with 3.0 silk suture using a figure-of-8 tie. The participant was discharged from the CTU with instructions for post procedure wound care.

### Flow cytometry: adipose tissue and blood

Inflammatory and anti-inflammatory cells were measured in AT and peripheral blood mononuclear cells (PBMCs) by flow cytometry. The immune cell populations isolated and quantified included M1 and M2 macrophages and monocytes, T regulatory (Treg) and T effector (Teff) lymphocytes. PBMCs were first isolated from whole blood by diluting the blood 1:1 in PBS and adding to SepMateTM-50 separation tubes (STEMCELL Technologies Inc., Vancouver, Canada) prefilled with 15 mL LymphoprepTM each (Axis-Shield, Oslo, Norway) and centrifugated at 1200 g for 15 min. Subcutaneous AT samples were weighed and digested in collagenase IV (200 U/mL, Worthington Biochemical Corporation) at 37 °C for 1 h and then processed on a 70 μm nylon cell strainer (Falcon®) into a single cell suspension. The stromal vascular fraction from the subcutaneous AT and the blood mononuclear cells were stained with the following surface marker antibodies: CD4, CD45, CD25, CD163, CD127, CD14. The following established flow cytometric gating strategies based on previous publications [42–47] were used to quantify the different immune cell sub-populations using the eight-color BD FACSCANTO analyzer, and data were acquired using BD FACSDiva software (BD Bioscience, San Jose, CA): CD45+, CD14+, CD163- for M1 macrophages, CD45+, CD14+, CD163+ for M2 macrophages, CD45+, CD4+, CD25+, CD127^low^ for regulatory T cells (Treg), CD45+, CD4+, CD25+, CD127^high^ for T effector cells (Teff). The total number of M1, M2, Treg, Teff subsets were quantified using FlowJo version 10 software (TreeStar Ashland OR) and were normalized to grams of AT collected or per milliliter of blood collected, respectively, as previously described [44, 47–49]. Gating strategy and flow cytometry of a representative sample are shown in Supplementary Fig. 1.

### Leptin and adiponectin

Leptin and adiponectin were measured using enzyme-linked immunosorbent assay (ELISA) kits (Millipore, Human Leptin dual range ELISA kit, Cat. # EZHL-80SK and Millipore Human Total Adiponectin ELISA kit, Cat. # EZHADP-61 K), and the ratio of leptin to adiponectin in serum, a functional marker of AT inflammation, insulin resistance and cardiometabolic risk, were calculated [50–55]. 10 uL of serum sample and lysate sample were diluted 1:50 and 1:500 for adiponectin measurements in serum and adipose lysate, respectively. For leptin, 25uL of serum sample and 50uL of lysate sample were diluted 1:4 or 1:10 and 1:2 for measurements of leptin in serum and adipose lysate, respectively. Samples were quantified in duplicate based on a standard curve for each adipokine and measured values were all within the limit of detection for each adipokine assay based on the range indicated by the manufacturer. The coefficients of variation (CV) for duplicate measurements of both adipokines were less than 10%. The intra-assay and inter-assay CV for leptin and adiponectin were 2.6 and 3.75% and 3.37 and 5.67%, respectively.

### Near-roadway and regional ambient air pollution exposures

Associations of adipokines with NRAP and ambient air pollution exposure during the year prior to biopsy were assessed. A residential address history was obtained at the time of enrollment. Residential addresses were standardized and their locations were geocoded using the Texas A&M Geocoder (http://geoservices.tamu.edu/Services/Geocode/).

Exposures to average NRAP from freeway and non-freeway sources during the year prior to biopsy were estimated at each participant’s residence using the California Line Source Dispersion Model (CALINE4). CALINE4 uses roadway geometry, traffic volumes, traffic counts, vehicle emission factors, and meteorological conditions including wind speed and direction, pollution mixing heights, and atmospheric stability as inputs to estimate the incremental ambient concentration contributed by vehicle emissions on local roadways [56]. The vehicle emission rates were obtained from the EMFAC2017 model for the vehicle mix and speeds on the different roadway classes [57]. The modeled NRAP exposures reflect the mixture of multiple pollutants from nearby traffic, such as particles and gases, including oxides of nitrogen (NOx), organic compounds, carbon monoxide, elemental carbon, and polycyclic aromatic hydrocarbons (PAH). The freeway, non-freeway and total NRAP mixtures were estimated as NOx in parts per billion (ppb).

Regional air pollution exposure levels for each participant were obtained from ambient monitoring stations by downloading hourly air quality data from the U.S. Environmental Protection Agency’s Air Quality System (AQS, http://www.epa.gov/ttn/airs/airsaqs). Hourly air quality data were summarized as 24-h average for particulate matter less than 2.5 um (PM_2.5_) and less than 10 um in aerodynamic diameter (PM_10_), nitrogen dioxide (NO_2_) and 8-h average daily maximum for ozone (O_3_). AQS air monitors stations in urban areas of southern California are spaced 20–30 km apart, which provide good characterization of the regional air pollution gradients across this region. The gaseous pollutants NO_2_ and O_3_ were measured using Federal Reference Method (FRM) continuous monitors; PM_2.5_ and PM_10_ were measured using FRM or Federal Equivalent Method (FEM) monitors. Monthly average values for each of the regional air pollutants were calculated from daily data with at least 75% completeness. To assign air pollution to the participant’s residential address, monthly average values were spatially interpolated from up to four monitoring stations within a 50 km radius of the participant’s residence, using an inverse distance-squared weighting (IDW2) algorithm, as previously described [58]. Prior work by our group has validated this approach for estimating monthly exposure [59]. However, when a participant’s home was located within 5 km of one or more monitoring stations, the interpolation was based solely on the values from the nearest monitor. Because ambient pollution estimates vary daily (unlike NRAP estimates), we also estimated exposures 1 month and 3 months prior to the day of the biopsy.

### Statistical analyses

We used multivariate linear regression models to examine the associations of leptin, adiponectin and the ratio of leptin to adiponectin in serum and AT with freeway, non-freeway and total NRAP exposure, and with regional PM_2.5_, PM_10_, NO_2_, and O_3_, during the year prior to biopsy. We also conducted sensitivity analyses using 1-month and 3-month average regional air pollution exposures prior to the day of biopsy. The linearity assumption of the models was checked and no violations were found.

For pollutants associated with adipokine, we fitted linear regression models including an interaction term between air pollutant and cell counts to examine the modification of the air pollution effect on adipokines by the cell counts. In these models, we categorized the cell counts (Treg, Teff, M1, and M2) and the ratios of M1 to M2 cell counts and Teff to Treg cell counts at the median value (> median = high and ≤ median = low) and included an air pollutant (continuous) × cell count or ratio (categorical) interaction term. Models were adjusted for age (continuous), sex (categorical), race/ethnicity (categorical). Smoking status as a categorical (never/ever smoker) was examined but was not identified as a confounder and was not included in final models. BMI confounded associations of air pollution with adipokines and was included in analyses, although it could be on the causal pathway between air pollution and metabolic dysregulation. For pollutant effects moderated by cell counts, we fitted logistic regression models to examine the main effects of pollutant on cell counts, the ratio of M1 to M2 cells counts and the ratio of Teff to Treg cells counts, each dichotomized as described above. We also fitted linear regression models to examine the main effects of cell counts and ratios (dichotomized as described above) on the adipokine associated with air pollution. Models were adjusted for the same covariates as the air pollution and adipokine analysis.

Results are presented as mean (standard deviation [SD]) or number (%), as appropriate. The estimates of the associations are reported as percentage changes in leptin and adiponectin levels per IQR increase in air pollution concentrations. A two-tailed *p*-value < 0.05 was considered statistically significant. Statistical analyses were conducted using SAS 9.4 (SAS Institute, Cary, NC) and R 3.5 (R Foundation for Statistical Computing, Vienna, Austria). The interaction plots were generated using the *ggeffects* package in R [60].

## Results

### Study population

Table 1 shows the characteristics of the 30 overweight (*N* = 4) or obese (*N* = 26) young adult participants. The mean age was 22.0 (SD 3.12; range 18–26) years. There were more female participants (60%) than male, and 73.3% of the study population was Hispanic. Each participant had complete serum adipokines and blood flow cytometry data. AT adipokines and flow cytometry were available for 29 participants.

**Table 1:** Descriptive statistics for demographic and clinical characteristic of participants

	Mean (SD) or N [%]
No. of participants	30
Age, years	22.0 (3.12)
**Sex**	
Women	18 [60.0%]
**Overweight/Obesity**	
Overweight (25 < BMI^a^ and < 30)	4 [13.3%]
Obese (BMI^a^ ≥ 30)	26 [86.7%]
**Race/Ethnicity**	
Hispanic	22 [73.3%]
**Smoking status**	
Never smoker	16 [55.2%]
Ever smoker	13 [44.8%]
**Blood Adipokines**	
Leptin (ng/ml)	64.8 (47.6)
Adiponectin (μg/ml)	9.3 (4.1)
Leptin/Adiponectin Ratio	8.7 (7.2)
**Adipose Tissue Adipokines**	
Leptin (ng/ml)	10.5 (6.5)
Adiponectin (μg/ml)	0.912 (0.373)
Leptin/Adiponectin Ratio	11.9 (5.82)

^a^BMI, body mass index

### Near-roadway and regional air pollution distributions and correlations

**Fig. 1:**
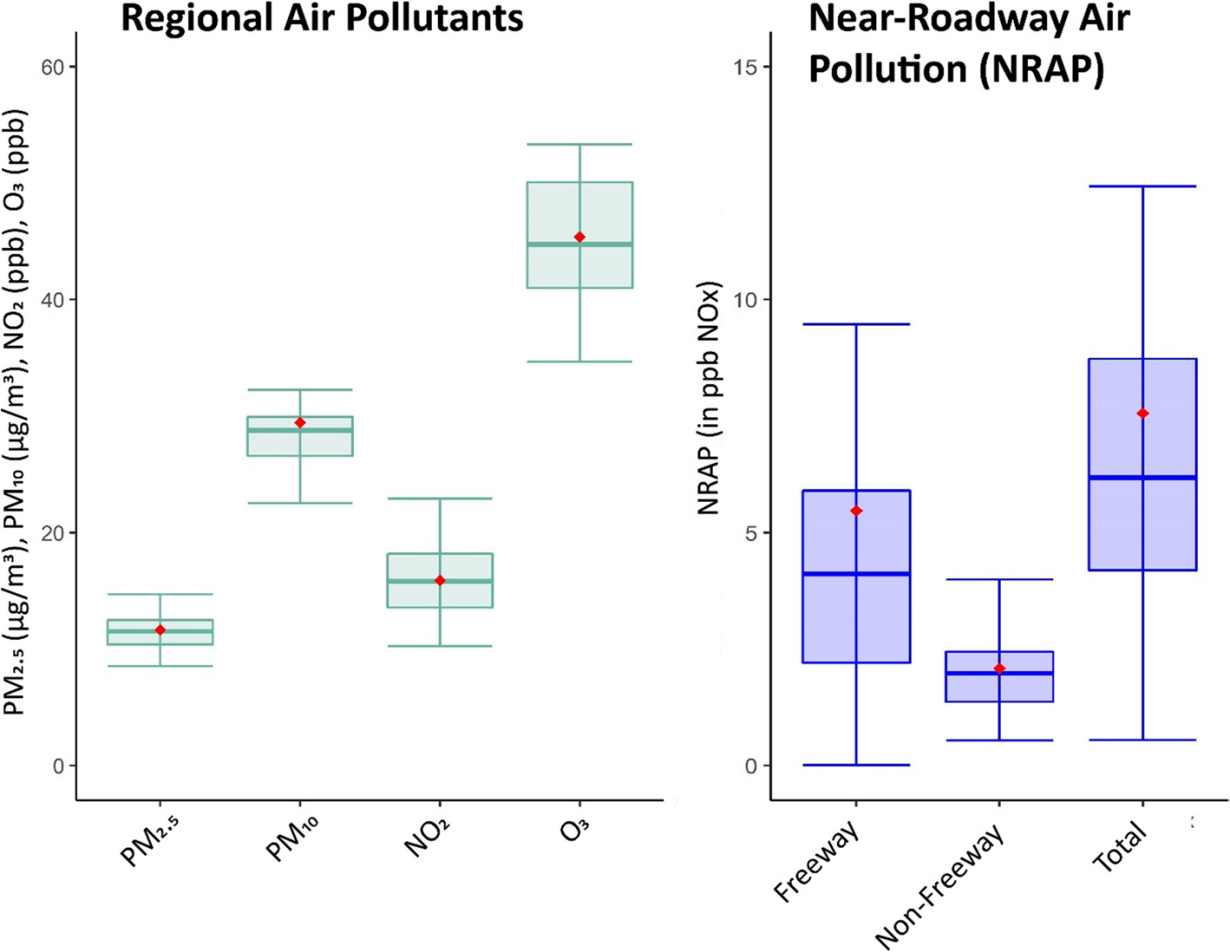
1-yr average regional and near-roadway ambient air pollution exposures prior to biopsy among 30 participants. The box shows the interquartile range, the line in the box is the median, and the red diamond is the mean

Figure 1 shows the distribution of regional and near-roadway air pollution exposures. The geometric mean concentrations during the year prior to biopsy of freeway, non-freeway, and total NRAP at the participants’ residential addresses were 5.5 ppb, 2.1 ppb, and 7.6 ppb, respectively, in CALINE NOx. Mean concentrations of regional PM_2.5_, PM_10_, NO_2_, and O_3_, were 11.6 μg/m^3^, 29.4 μg/m^3^, 15.9 ppb, and 45.4 ppb, respectively. Correlations of air pollution exposures were less than 0.50 except PM_2.5_ with PM_10_ (*r* = 0.54), NO_2_ with O_3_ (*r* = − 0.78) and freeway with total near-roadway air pollution (*r* = 0.99) (Supplementary Table 1).

### Adipokines in serum and adipose tissue

Leptin and adiponectin concentrations were higher in serum than in AT (Table 1), consistent with a previous study [61]. Leptin was positively correlated with adiponectin (*r* = 0.61) in AT but not in serum (Supplementary Fig. 2). Moderate to strong correlations were found between serum and AT adipokines (*r* = 0.65 for leptin, *r* = 0.47 for adiponectin, and *r* = 0.81 for leptin/adiponectin ratio; Supplementary Fig. 3), which did not appreciably change after controlling for BMI (*r* = 0.59 for leptin, *r* = 0.47 for adiponectin, and *r* = 0.72 for the leptin/adiponectin ratio) (data not shown). Serum leptin (*r* = 0.63) and the ratio of serum leptin/adiponectin (*r* = 0.64) were positively correlated with BMI (Supplementary Fig. 4) whereas an inverse correlation was observed between serum adiponectin and BMI (*r* = − 0.29). In AT, leptin (*r* = 0.35) and ratio of leptin/adiponectin (*r* = 0.54), but not adiponectin, were also positively correlated with BMI.

### Cell counts in blood and adipose tissue

The distribution of type 1 and 2 macrophages and Teff and Treg cells in blood and in AT are shown in Supplementary Table 2. Cell counts were higher in blood than in AT. The median counts of Treg, Teff, M1, and M2 cells in blood were 21,654, 24,840, 7959, and 64,427 cells/ml, respectively, and 3806, 9092, 4687, and 8197 cells/g in AT, respectively.

### Association of NRAP and regional air pollution exposures with adipokines in serum and adipose tissue

NRAP from non-freeway sources was positively associated with leptin in serum and AT. An IQR increase in past year non-freeway NRAP was associated with 31.7% (95% CI: 10.4, 52.9%) higher leptin concentration in serum and 19.4% (95% CI: 2.2, 36.6%) higher leptin level in AT, after adjusting for age, sex, race, and ethnicity (Table 2). Although adiponectin level itself was not associated with NRAP exposure, the ratio of leptin/adiponectin in serum was associated with non-freeway NRAP [(27.9% (95% CI: 0.33, 55.4%) per IQR increase in non-freeway NOx]. When the models were further adjusted for BMI, the association of non-freeway NRAP with serum leptin was attenuated but still significant; the association with AT leptin became marginally significant (*p* = 0.09) (Supplementary Table 3). The attenuation of effect estimates after adjusting for BMI may reflect confounding by BMI or inappropriate inclusion of a variable on the causal pathway. No associations of adipokines with regional air pollution exposures were found. In sensitivity analyses, 1-month and 3-month average regional air pollution exposures prior to the day of the biopsy also were not associated with adipokine levels in serum or AT (results not shown).

**Table 2:** Association between Near-roadway and Regional Ambient Air Pollution Exposures and Adipokines in Blood and in Tissue. Results were scaled to an interquartile increase in PM_2.5_ (2.2 μg/m^3^), PM_10_ (3.5 μg/m^3^), NO_2_ (4.8 ppb), O_3_ (9.3 ppb), freeway NRAP (3.8 ppb NOx), non-freeway NRAP (1.1 ppb NOx), and total NRAP (4.8 ppb NOx)

Model was adjusted for age, sex, and race/ethnicityModel	Serum Adipokines			Adipose Tissue Adipokines		
Near-Roadway Air Pollutants	Leptin % Change (95% CI)	Adiponectin % Change (95% CI)	Leptin/adiponectin Ratio % Change (95% CI)	Leptin % Change (95% CI)	Adiponectin % Change (95% CI)	Leptin/adiponectin Ratio % Change (95% CI)
Freeway	3.96 (−8.58, 16.5)	3.8 (−3.84, 11.5)	0.30 (−14.8, 15.4)	−0.44 (−10.1, 9.22)	0.30 (−6.34, 6.93)	0.37 (− 8.16, 8.91)
Non-freeway	**31.7 (10.4, 52.9)**	−5.36 (−20.5, 9.8)	**27.9 (0.33, 55.4)**	**19.4 (2.15, 36.6)**	5.28 (−7.56, 18.1)	9.45 (−6.83, 25.7)
Total	7.88 (−7.67, 23.4)	4.25 (−5.40, 13.9)	2.90 (−16.0, 21.8)	1.26 (−10.9, 13.4)	0.86 (−7.46, 9.19)	1.34 (−9.36, 12.1)
**Regional Air Pollutants**						
PM_2.5_	6.30 (−33.2, 45.8)	−3.52 (−27.9, 20.9)	25.6 (− 20.5, 71.7)	−6.24 (− 37.9, 25.4)	4.06 (−17.7, 25.8)	−3.55 (− 31.6, 24.5)
PM_10_	−7.72 (− 22.9, 7.40)	−5.34 (−14.6, 3.94)	−2.63 (− 21.0, 15.8)	−10.4 (− 21.9, 1.04)	− 5.45 (−13.5, 2.64)	−5.04 (−15.6, 5.54)
NO_2_	−5.31 (−46.6, 36.0)	−5.75 (− 31.2, 19.7)	10.8 (−38.3, 59.9)	−0.18 (− 31.8, 31.4)	−3.32 (− 25.0, 18.3)	14.6 (−12.7, 41.8)
O_3_	−2.92 (− 52.3, 46.5)	1.62 (− 28.9, 32.1)	− 11.3 (− 70.1, 47.4)	−21.2 (− 59.9, 17.6)	0.04 (−27.2, 27.3)	−32.1 (− 64.6, 0.44)

### NRAP associations with leptin modified by cell counts

In serum, the non-freeway NRAP association with serum leptin was larger for participants in the upper half of the pro-inflammatory Teff/Treg ratio distribution compared with participants with lower T-cell ratio, after adjusting for age, sex, race, ethnicity, and BMI (Supplementary Table 4). There were also significant interactions between NRAP effect estimates among those with greater pro-inflammatory M1 cell counts in AT (compared with effects in low cell counts) and among those with greater M1/M2 cell ratios in serum (compared with effects in low cell counts). In AT, non-freeway NRAP associations with leptin were larger among participants with higher M1 cell counts than with lower cell counts; this effect estimate was little changed after further adjusting for complementary M2 cell counts (results not shown). No other differences in the non-freeway NRAP effects by immune cell distribution were observed.

**Fig. 2:**
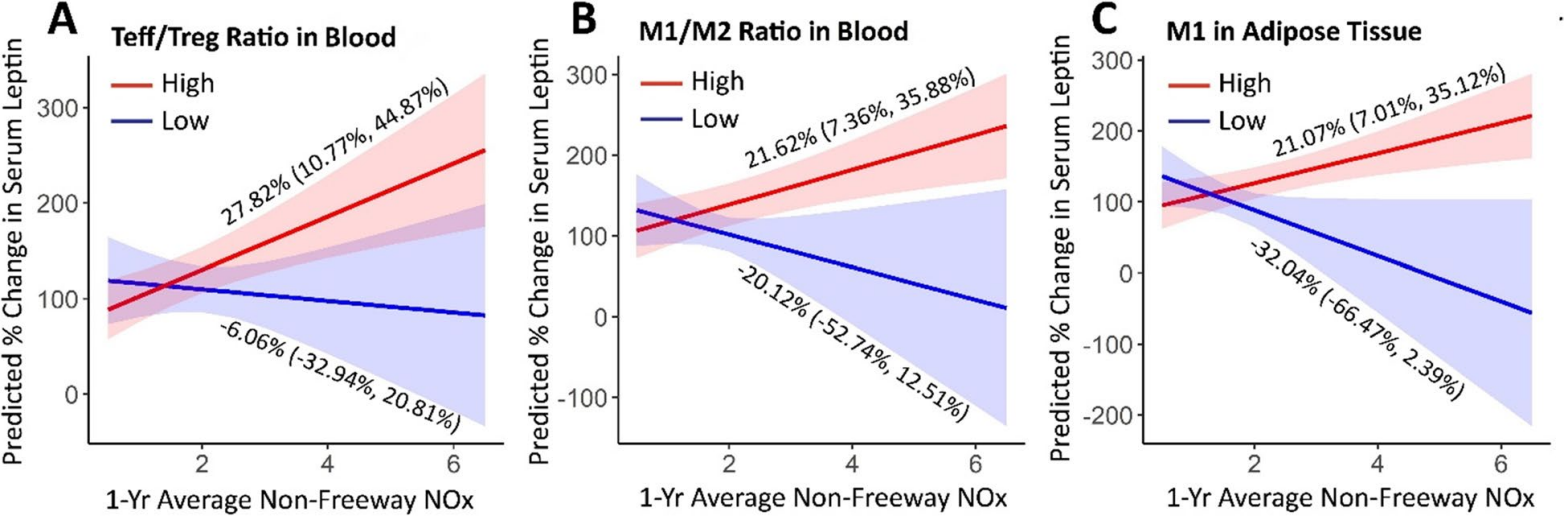
Stratum-specific effects of 1-yr average non-freeway NRAP by **A**) the ratio of Teff to Treg cells counts in serum, **B**) the ratio of M1 to M2 cells counts in serum, **C**) M1 cell counts in adipose tissue. The ratio of Teff to Treg in serum, M1 to M2 in serum, and M1 in adipose tissue were dichotomized at the median of 1.10, 0.14, and 4687 cells/g, respectively (Supplementary Table 2). The lighter color indicates the confidence intervals. The reported effect estimates were scaled to an interquartile (1.1 ppb) increase in non-freeway NOx. All the models were adjusted for age, sex, race, ethnicity, and BMI

The stratum-specific effects for these significant interactions are shown in Fig. 2. Non-freeway NRAP was associated with a 27.8% increase in serum leptin per 1.1 ppb CALINE NOx (95% CI: 10.8, 44.9%) in participants with high blood Teff /Treg cell ratio and with a 6.06% decrease in serum leptin (95% CI: − 32.9, 20.8%) in those with a low ratio. The non-freeway effect estimate in those with high blood M1/M2 ratio was 21.6% (95% CI: 7.36, 35.9%) compared with − 20.1% (95% CI: − 52.7, 12.5%) in those in the lower half of the M1/M2 distribution. In AT, non-freeway NRAP was associated with a 21.1% increase in serum leptin per 1.1 ppb CALINE NOx (95% CI: 7.01, 35.1%) in those with greater AT M1 cell counts and with a 32.0% decline (95% CI: − 66.5, 2.39) in those with lower M1 counts. Thus, the larger NRAP associations with leptin in a greater pro-inflammatory milieu were driven in part by negative associations with leptin in the less inflammatory immune environment.

### NRAP associations with cell counts

We did not find associations between non-freeway NRAP and high cell counts, the ratio of M1 to M2 cells counts, or the ratio of Teff to Treg cells counts in AT (Supplementary Table 5). In blood, non-freeway NRAP was associated with higher odds of high Treg counts.

### Cell counts association with leptin in serum

We found a significant association between higher M1 cell counts in AT and increased leptin in serum. However, we did not find associations between serum leptin and high cell counts, the ratio of M1 to M2 cells counts, or the ratio of Teff to Treg cells counts in blood (Supplementary Table 6).

## Discussion

Novel findings from this study include the associations of non-freeway but not freeway NRAP with leptin levels and with the ratio of leptin to adiponectin, which are functional measures of AT inflammation and insulin resistance [50–55], in blood or AT. The associations of non-freeway NRAP with serum leptin were observed only if there were increased Teff/Treg or M1/M2 ratios in blood or if there were elevated M1 cell concentrations in AT. Thus, a proinflammatory cellular environment in either blood or AT was associated with a stronger effect of non-freeway NRAP, and the more relevant tissue for identifying inflammatory adipokine effects was blood, as no associations with AT adipokines were found. There were no associations between regional air pollution exposures and adipokines levels.

This is one of the few studies to distinguish effects of NRAP from non-freeway and freeway sources. One previous study also showed serum leptin (in cord blood) was associated with non-freeway but not with freeway NRAP [22]. A few other studies, including studies from our group, have also shown stronger non-freeway associations with BMI [18, 62] and other outcomes [63–67]. Possible reasons for larger effect of non-freeway NRAP are that non-freeway roads are generally in much closer proximity to homes, such that highly reactive components in the tailpipe mixture, if responsible for these effects, may not be present at typical distances of homes to freeways. Non-freeway NRAP also may contain more non-combustion brake wear and tire wear particles resulting from frequent stops and accelerations. A recent study showed that brake abrasion dust and diesel exhaust particles have similar toxicity to human macrophage function [68]. Cold starts, which are more likely to occur in residential areas, result in higher concentration of NRAP [69]. It is also possible that non-freeway NRAP exposure captures other neighborhood (e.g. housing and built environment) characteristics responsible for residual confounding.

Gasoline spark ignition (SI) engines are the primary combustion source of non-freeway NRAP in Southern California. Non-freeway roadways have very little heavy duty truck traffic with diesel compression ignition (CI) engines that are largely restricted to freeways. Therefore, although the reason non-freeway NRAP was more strongly associated with leptin concentrations is not clear, the results indicate that diesel exhaust was *not* responsible for these effects. One Southern California study showed that the chemical composition of gasoline SI engine emissions had greater amounts of high-molecular-weight particulate polyaromatic hydrocarbons (PAHs) than did emissions from CI engines more common on freeways [70]. A Texas study showed that numerous PAHs, acetaldehyde, formaldehyde, and acrolein were present in higher concentrations 35 m downwind of a major non-freeway roadway than 40 m downwind of a freeway [71]. There has been little toxicological study focused on vehicular emissions from gasoline engines; most vehicular combustion toxicology has been focused on diesel exhaust, especially effects of particulate in diesel exhaust. One study with source-apportioned PM_2.5_ in Atlanta, Georgia, a major urban population center which like Southern California has relatively high emissions of PM_2.5_ from traffic sources, reported that PM_2.5_ from light-duty gasoline vehicles exhibited the highest oxidative potential, followed by biomass burning, and by heavy-duty diesel vehicles [72]. Thus, the toxicity and health effects of gasoline combustion emissions merit additional study.

This was the first epidemiological study to examine the role of macrophages and T-cells as modifiers of NRAP effects on adipokines. Emerging data from animal and human studies suggest that T cell counts in blood modulate AT inflammation and insulin resistance [47, 73, 74]. The increased NRAP-associated serum leptin in the presence of increased Teff/Treg ratio in blood is consistent with a proinflammatory role of effector T cells. A prior in vitro study, showing that regulatory T cells reduced air pollution-induced inflammatory responses and NF-κB activation, is also generally consistent with immune activation of inflammatory pathways in association with air pollution exposure [75]. Macrophages are the main source of inflammatory mediators in AT of obese subjects [76–79]. Our results suggest that increased M1 (inflammatory) macrophage concentration in AT increased the inflammatory effect of NRAP on leptin. We also found that M1 macrophage polarization (e.g., higher proportion of M1 compared to M2 (anti-inflammatory cells) in blood, but not in AT, was associated with increased NRAP estimated effects on serum leptin. This is consistent with in vitro studies showing that infiltration of effector T cells or loss of protective regulatory T cells led to macrophage recruitment and differentiation, which enhanced AT inflammation and systemic insulin resistance [80]. We also found an association between higher M1 cell counts in AT and increased leptin in serum, consistent with interaction model results showing that higher pro-inflammatory M1 cell counts in AT moderated the non-freeway NRAP associations with serum leptin. There were no associations of other immune cell profiles in AT or in blood with leptin in serum. We observed no associations of non-freeway NRAP with cells in AT; in blood non-freeway NRAP was associated only with higher regulatory T cells counts. The biological plausibility of this association is not clear. This is a cross-sectional study; it is possible that the higher regulatory T cells counts in blood resulted from a compensatory response to other inflammatory effects of non-freeway NRAP. These associations of NRAP with cell types merit further study.

We observed positive correlations between leptin and adiponectin in AT, which was unexpected, as previous studies have reported inverse correlations between leptin and adiponectin in blood [81–83]. To our knowledge, no previous study has examined the relationship between leptin and adiponectin in AT. Therefore, we examined mRNA level associations of leptin and adiponectin in AT using publicly available gene expression data from the GTEx Project [84], and we found positive associations (Supplementary Fig. 5). The relevance of these observations to the absence in our study of associations between AT adipokine levels and air pollution remains to be determined.

Strengths of this study include a unique study population of overweight/obese young adults at risk for developing metabolic disease, from whom both adipose and blood samples were available. This allowed us to identify novel associations of adipokines specifically with non-freeway NRAP and to explore the role of pro-inflammatory T cell and macrophage profiles in these associations. The generally consistent associations with of serum leptin with NRAP in blood and AT suggest that blood may be a good proxy for effects in the AT. We also acknowledge some limitations. The analysis was cross-sectional, limiting the directional interpretation of these relationships. We conducted multiple testing for associations of several air pollution exposures with leptin; however, the association between NRAP and serum leptin level was significant even after Bonferroni correction. Information about time spent at home and outdoors was not available. However, measurement error due to time away from home is likely to be non-differential with respect to adipokine concentrations, and time outside exercising might increase both inhaled dose of air pollution and reduce leptin; therefore, observed effects of NRAP may have underestimated the true associations. We did not have data on participant physical activity, which might have increased the dose of air pollution. However, the association between air pollution exposure and physical activity, and the combined effect on health, is inconclusive [85]; a randomized controlled study reported no association between exercise and leptin level [86]. The study population was overweight and obese, and young adult, so the findings from this study may not be generalizable to normal weight and/or lean populations, or to other age groups. Given the small study sample size, larger and prospective studies will be needed to address new research questions raised by our observations.

## Conclusion

The results of this study have potential policy implications. A common wisdom is that diesel engine exhaust is the more toxic component of traffic related air pollution [85–87]. However, the association of leptin with non-freeway NRAP suggests that ubiquitous gasoline exhaust merits more toxicological study to assess metabolic and immune modulating effects, potentially leading to regulation of NRAP. Further prospective studies are needed to better understand biological mechanisms underlying metabolic effects of NRAP and of moderating immune cell profiles.

## Supplementary Information

**Additional file 1.** Supplementary Material.

## Abbreviations

AT: Adipose Tissue
BMI: Body Mass Index
M1: Pro-Inflammatory Macrophage
M2: Anti-Inflammatory Macrophage
NOx: Oxides of Nitrogen
NRAP: Near-Roadway Air Pollution
Teff: Effector T cells
Treg: Regulatory T cells
