# Supplementary material for: Near-roadway air pollution, immune cells and adipokines among obese young adults
Source: Environ Health. 2022 Mar 19;21:36. doi: 10.1186/s12940-022-00842-2 (PMC8933931; doi:10.1186/s12940-022-00842-2)
Supplement: Supplementary file 1 — Additional file 1. Supplementary Material. [file 12940_2022_842_MOESM1_ESM.docx]

**Supplemental Material**

**Near-Roadway Air Pollution, Immune Cells and Adipokines among Obese Young Adults**

Md Mostafijur Rahman^1*^, Fei Fei Liu^1^, Sandrah P. Eckel^1^, Ishwarya Sankaranarayanan^2^, [Pedram Shafiei-Jahani](https://urldefense.com/v3/__https:/www.nature.com/articles/s41467-021-22832-7*auth-Pedram-Shafiei_Jahani__;Iw!!LIr3w8kk_Xxm!9A0U50Jtz9_Yl7Kupf-Gg5fOU2iZ9bZhm29r_nvBfR3jaz0yHQ9CueJ5RFDjnyElbht3$)^2^, Emily Howard^2^, Lilit Baronikian^3^, Fred Sattler^3^, Frederick W. Lurmann^4^, Hooman Allayee^1^, Omid Akbari^2^, and Rob McConnell^1^

^1^Department of Preventive Medicine, Keck School of Medicine, University of Southern California, Los Angeles, CA, USA

^2^Department of Molecular and Cellular Immunology, University of Southern California, Los Angeles, California, USA

^3^Department of Medicine, Keck School of Medicine, University of Southern California, Keck School of Medicine, Los Angeles, CA, USA

^4^Sonoma Technology, Inc., Petaluma, CA, USA

***Corresponding Author:**

Rob McConnell, MD

Department of Preventive Medicine,

Keck School of Medicine, University of Southern California

2001 N. Soto Street Building: SSB Los Angeles, CA 90032, USA

Email: [rmcconne@usc.edu](mailto:rmcconne@usc.edu)

Supplementary Table 1. Pearson correlations among near-roadway and regional air pollutants

|  | **Freeway NOx** | **Non-Freeway NOx** | **Total NOx** | **PM_2.5_** | **PM_10_** | **NO_2_** | **O_3_** |
| --- | --- | --- | --- | --- | --- | --- | --- |
| **Freeway NOx** | 1 |  |  |  |  |  |  |
| **Non-Freeway NOx** | 0.04 | 1 |  |  |  |  |  |
| **Total NOx** | 0.99 | 0.18 | 1 |  |  |  |  |
| **PM_2.5_** | -0.05 | -0.12 | -0.07 | 1 |  |  |  |
| **PM_10_** | -0.16 | -0.31 | -0.2 | 0.54 | 1 |  |  |
| **NO_2_** | 0.25 | 0.05 | 0.25 | -0.11 | 0.11 | 1 |  |
| **O_3_** | -0.33 | -0.12 | -0.34 | 0.42 | 0.31 | -0.78 | 1 |

Supplementary Table 2. Distribution of blood and adipose tissue cell counts

|  |  | **Median** | **IQR (Q1, Q3)** | **Range (Max-Min)** |
| --- | --- | --- | --- | --- |
| **Blood Cell Counts (cells/ml)** | Teff | 24840 | 17491 - 33719 | 6028 - 138807 |
|  | Treg | 21654 | 14590 - 33163 | 6329 - 84821 |
|  | Teff/Treg | 1.10 | 0.70 – 1.58 | 0.16 – 5.32 |
|  | M1 | 7959 | 3091 - 17665 | 1067 - 32494 |
|  | M2 | 64427 | 18512 - 100140 | 5185 - 191563 |
|  | M1/M2 | 0.14 | 0.11 – 0.22 | 0.03 – 0.45 |
|  |  |  |  |  |
| **Adipose Tissue Cell Counts (cells/g)** | Teff | 9092 | 3438 - 15609 | 498 - 62331 |
|  | Treg | 3806 | 1262 - 9108 | 458 - 38325 |
|  | Teff/Treg | 3.07 | 0.83 – 5.97 | 0.30 – 14.5 |
|  | M1 | 4687 | 3651 - 8035 | 562 - 38805 |
|  | M2 | 8197 | 3073 - 14536 | 624 - 274835 |
|  | M1/M2 | 0.67 | 0.48 – 0.91 | 0.08 – 2.70 |

Treg, regulatory T cells; Teff, effectors T cells; M1, M1 macrophages; M2, M2 macrophages;

IQR, interquartile range, Q1, 1^st^ quartile; Q3, 3^rd^ quartile.

| **Model** | **Serum Adipokines** | | |  | **Adipose Tissue Adipokines** | | |
| --- | --- | --- | --- | --- | --- | --- | --- |
| **Near-Roadway Air Pollutants** | **Leptin % Change (95% CI)** | **Adiponectin % Change (95% CI)** | **Leptin/adiponectin Ratio % Change (95% CI)** |  | **Leptin % Change (95% CI)** | **Adiponectin % Change (95% CI)** | **Leptin/adiponectin Ratio % Change (95% CI)** |
| Freeway | -3.45 (-11.4, 4.49) | 5.84 (-1.75, 13.4) | -8.32 (-18.4, 1.74) |  | -3.71 (-12.8, 5.40) | 0.84 (-6.20, 7.87) | -3.70 (-10.6, 3.15) |
| Non-freeway | **17.0 (2.19, 31.9)** | -1.83 (-17.8, 14.1) | 9.06 (-11.9, 30.0) |  | 15.5 (-2.69, 33.8) | 7.30 (-6.50, 21.1) | 0.39 (-13.7, 14.5) |
| Total | -2.90 (-13.1, 7.31) | 7.34 (-2.37, 17.1) | -9.85 (-22.8, 3.08) |  | -3.50 (-15.2, 8.20) | 1.75 (-7.21, 10.7) | -4.72 (-13.5, 4.02) |
| **Regional Air Pollutants** |  |  |  |  |  |  |  |
| PM_2.5_ | -0.36 (-24.7, 23.9) | -2.08 (-26.1, 21.9) | 18.3 (-12.8, 49.4) |  | -8.87 (-37.8, 20.1) | 4.49 (-17.7, 26.7) | -6.86 (-28.9, 15.1) |
| PM_10_ | -2.92 (-12.4, 6.56) | -6.58 (-15.6, 2.48) | 2.88 (-9.67, 15.4) |  | -8.35 (-19.2, 2.46) | -5.99 (-14.3, 2.30) | -2.18 (-10.8, 6.41) |
| NO_2_ | -16.8 (-41.3, 7.83) | -3.37 (-28.6, 21.9) | -1.62 (-35.2, 32.0) |  | -4.91 (-34.1, 24.2) | -2.64 (-24.9, 19.6) | 8.81 (-13.1, 30.7) |
| O_3_ | 15.8 (-14.3, 45.9) | -2.45 (-32.8, 27.9) | 9.26 (-31.1, 49.6) |  | -12.8 (-49.6, 24.0) | -1.43 (-29.7, 26.9) | -21.7 (-48.4, 5.04) |

Supplementary Table 3. Association between Near-roadway and Regional Ambient Air Pollution Exposures and Adipokines in Blood and Tissue

Results were scaled to an interquartile increase in PM_2.5_ (2.2 µg/m^3^), PM_10_ (3.5 µg/m^3^), NO_2_ (4.8 ppb), O_3_ (9.3 ppb), freeway NRAP (3.8 ppb NOx), non-freeway NRAP (1.1 ppb NOx), and total NRAP (4.8 ppb NOx). Model was adjusted for age, sex, and race/ethnicity, and BMI.

Supplementary Table 4. Associations of Non-Freeway Near-roadway Air Pollutant (NRAP) Exposure with Serum and Adipose Tissue Leptin in High Cell Count Strata (Compared with Low Cell Count Reference Group)

|  | **Joint Effect*** | **Serum Leptin % Change NRAP effect estimate (as percent change) (95% CI)** | **p-interaction** | **Tissue Leptin % Change NRAP effect estimate (as percent change) (95% CI)** | **p- interaction** |
| --- | --- | --- | --- | --- | --- |
| **Cell Counts in Blood** | Non-Freeway NOx |  |  |  |  |
|  | Treg Low | Reference |  | Reference |  |
|  | Treg High | 6.67 (-36.5, 49.9) | 0.75 | 18.5 (-35.8, 72.9) | 0.49 |
|  | Teff Low | Reference |  | Reference |  |
|  | Teff High | 12.0 (-24.2, 48.1) | 0.50 | 20.2 (-21.7, 62.2) | 0.33 |
|  | Teff/Treg Low | Reference |  | Reference |  |
|  | Teff/Treg High | **33.9 (0.97, 66.8)** | **0.04** | 18.6 (-23.5, 60.7) | 0.36 |
|  | M1 Low | Reference |  | Reference |  |
|  | M1 High | -15.4 (-52.6, 21.8) | 0.40 | -30.9 (-74.8, 12.9) | 0.16 |
|  | M2 Low | Reference |  | Reference |  |
|  | M2 High | -24.0 (-61.5, 13.6) | 0.20 | -40.6 (-83.5, 2.28) | 0.06 |
|  | M1/M2 Low | Reference |  | Reference |  |
|  | M1/M2 High | **41.7 (6.4, 77.1)** | **0.02** | 31.7 (-14.6, 78.0) | 0.17 |
|  |  |  |  |  |  |
|  | Treg Low | Reference |  | Reference |  |
| **Cell Counts in Tissue** | Treg High | 29.3 (-16.0, 74.6) | 0.19 | 33.1 (-22.6, 88.8) | 0.23 |
|  | Teff Low | Reference |  | Reference |  |
|  | Teff High | 35.4 (-5.2, 76.0) | 0.08 | 13.6 (-38.6, 65.9) | 0.59 |
|  | Teff/Treg Low | Reference |  | Reference |  |
|  | Teff/Treg High | -16.3 (-65.0, 32.4) | 0.40 | -29.1 (-85.2, 27.0) | 0.19 |
|  | M1 Low | Reference |  | Reference |  |
|  | M1 High | **53.1 (15.8, 90.5)** | **0.007** | 10.3 (-47.5, 68.0) | 0.71 |
|  | M2 Low | Reference |  | Reference |  |
|  | M2 High | 28.7 (-17.6, 74.9) | 0.21 | 17.6 (-42.0, 77.1) | 0.54 |
|  | M1/M2 Low | Reference |  | Reference |  |
|  | M1/M2 High | -16.6 (-53.4, 20.2) | 0.36 | -29.2 (-69.6, 11.2) | 0.14 |

Model was adjusted for age, sex, race/ethnicity, and BMI. Results were scaled to interquartile increase in non-freeway NRAP (1.1 ppb CALINE NOx).

* Joint effect was estimated by adding a multiplicative interaction term between continuous non-freeway NOx and dichotomized cell counts (or ratio) at the median into high and low strata (from supplementary Table 2

Supplementary Table 5. Associations (e.g., Odds Ratios) of Non-Freeway Near-roadway Air Pollutant (NRAP) Exposure with Cell Counts, the Ratio of M1 to M2 Cells Counts and the Ratio of Teff to Treg Cells Counts.

|  | **Cell counts in Tissue** | | | | | | |
| --- | --- | --- | --- | --- | --- | --- | --- |
| Pollutant | Teff | Treg | Teff/Treg ratio |  | M1 | M2 | M1/M2 ratio |
| Non-Freeway NOx | 2.05 (0.90, 5.42) | 2.29 (0.91, 8.33) | 1.35 (0.64, 3.49) |  | 1.70 (0.82, 4.44) | 1.50 (0.75, 3.00) | 0.53 (0.17, 1.16) |
|  | **Cell counts in Blood** | | | | | | |
| Non-Freeway NOx | 1.11 (0.55, 2.66) | 3.35 (1.14, 16.11) | 0.86 (0.43, 1.86) |  | 0.84 (0.33, 1.75) | 1.12 (0.54, 2.77) | 2.89 (0.99, 12.15) |

Models were adjusted for for age, sex, race/ethnicity, and BMI. Results were scaled to interquartile increase in non-freeway NRAP (1.1 ppb CALINE NOx)

Supplementary Table 6. Associations of Higher Cell Counts or the Higher Ratio of M1 to M2 Cells Counts or the Higher Ratio of Teff to Treg Cells Counts (all dichotomized at the median) with Serum Leptin

|  |  | **Serum Leptin**  **(as percent change) (95% CI)** |
| --- | --- | --- |
| **Cell Counts in Blood** | Teff High | 9.13 (-26.09, 44.36) |
|  | Treg High | -0.77 (-36.08, 34.54) |
|  | Teff/Treg High | 11.94 (-21.8, 45.68) |
|  | M1 High | 9.51 (-27.82, 46.85) |
|  | M2 High | -0.77 (-37.51, 35.97) |
|  | M1/M2 High | -30.72 (-204.14, 142.7) |
|  |  |  |
| **Cell Counts in Tissue** | Teff High | 22.81 (-15.92, 61.54) |
|  | Treg High | 8.93 (-26.2, 44.06) |
|  | Teff/Treg High | 21.83 (-18.6, 62.26) |
|  | M1 High | **36.16 (5.85, 66.48)** |
|  | M2 High | 17.99 (-14.69, 50.67) |
|  | M1/M2 High | -5.59 (-36.67, 25.49) |

The cell counts (Treg, Teff, M1, and M2) and the ratios of M1 to M2 cell counts and Teff to Treg cell counts were categorized at the median value (> median = High and ≤ median = Low). Models were adjusted for for age, sex, race/ethnicity, and BMI.


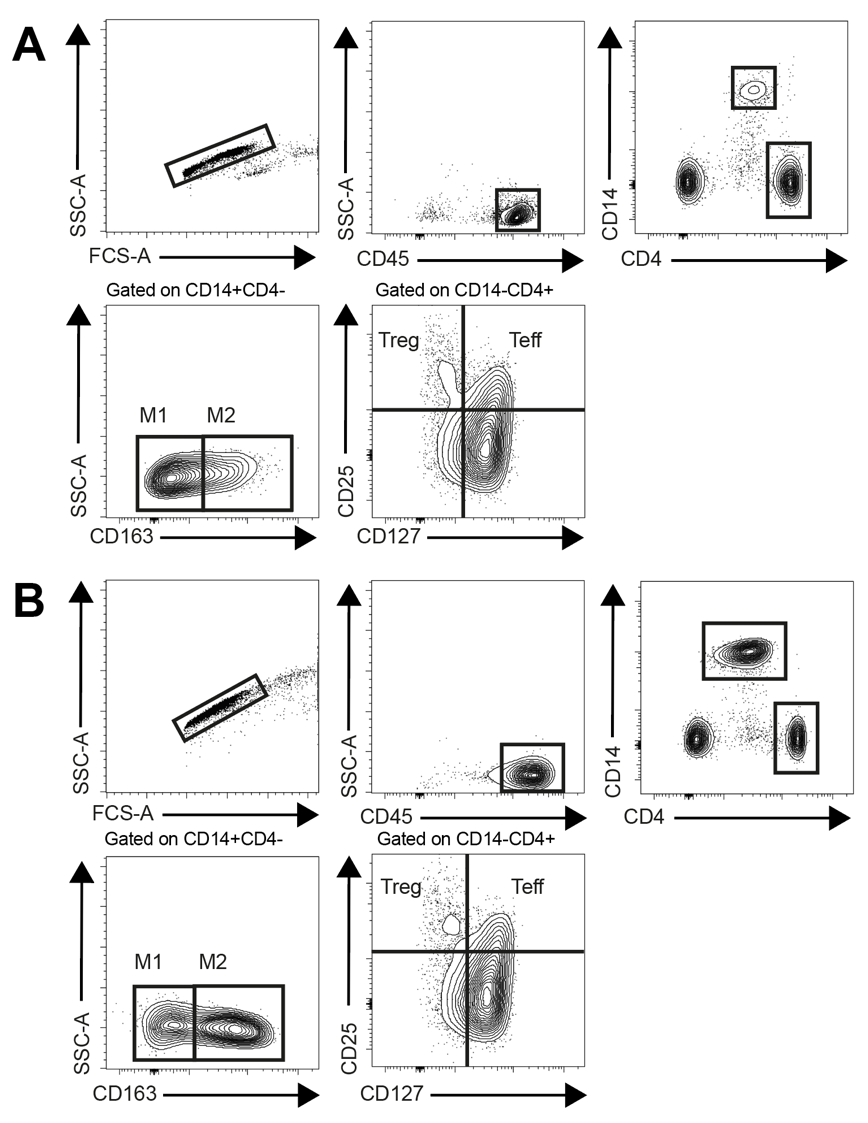


Supplementary Figure 1. Gating strategies for human blood and adipose tissue. Gating strategy of Tregs, Teffs, M1 and M2 cells in blood (A) and in adipose tissue (B). Tregs and Teffs are both positive for CD45, CD4 and CD25. Additionally, while Tregs are CD127-, Teffs are CD127+. M1 and M2 cells are gated as positive for CD45 and CD14. M1 cells are identified as CD163- whereas M2 cells are CD163+.


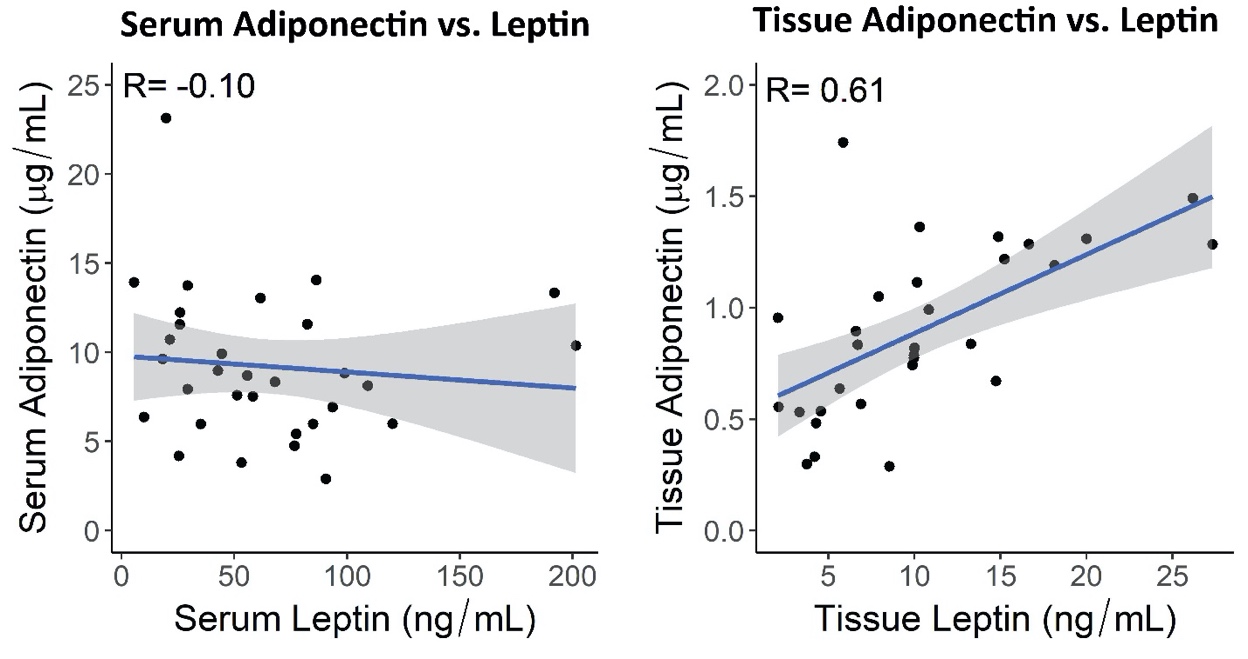


Supplementary Figure 2. Correlation between adiponectin and leptin in serum and in adipose tissue.


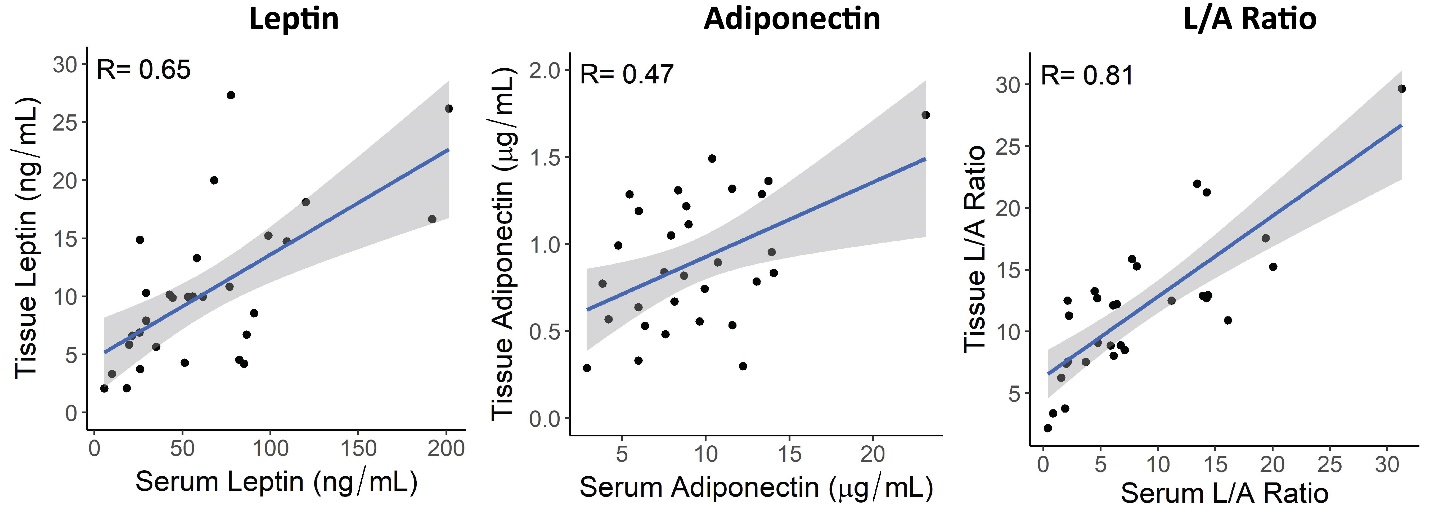


Supplementary Figure 3. Correlation between tissue and serum leptin, adiponectin, and leptin/adiponectin ratio.


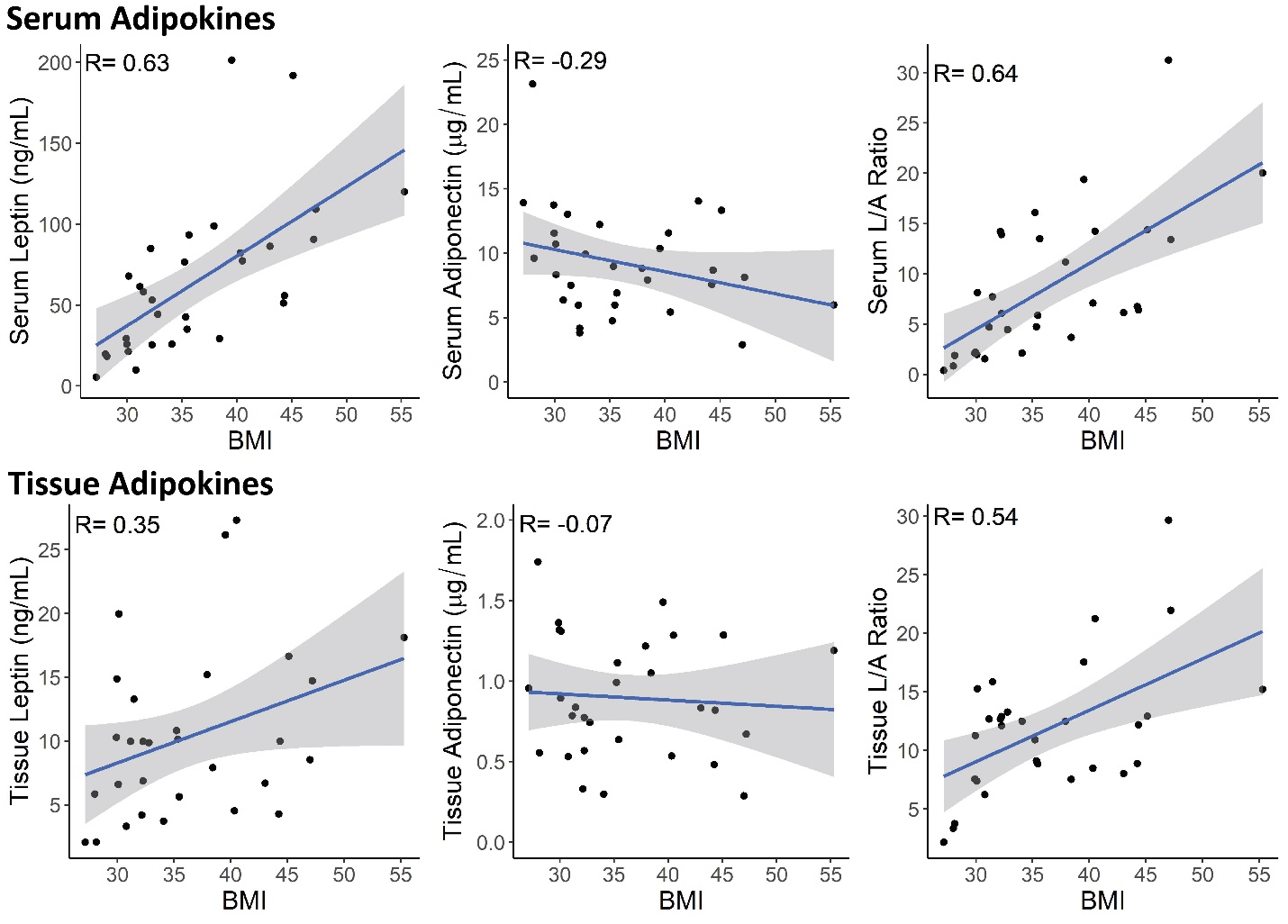


Supplementary Figure 4. Correlations between BMI and leptin and adiponectin in serum and adipose tissue.


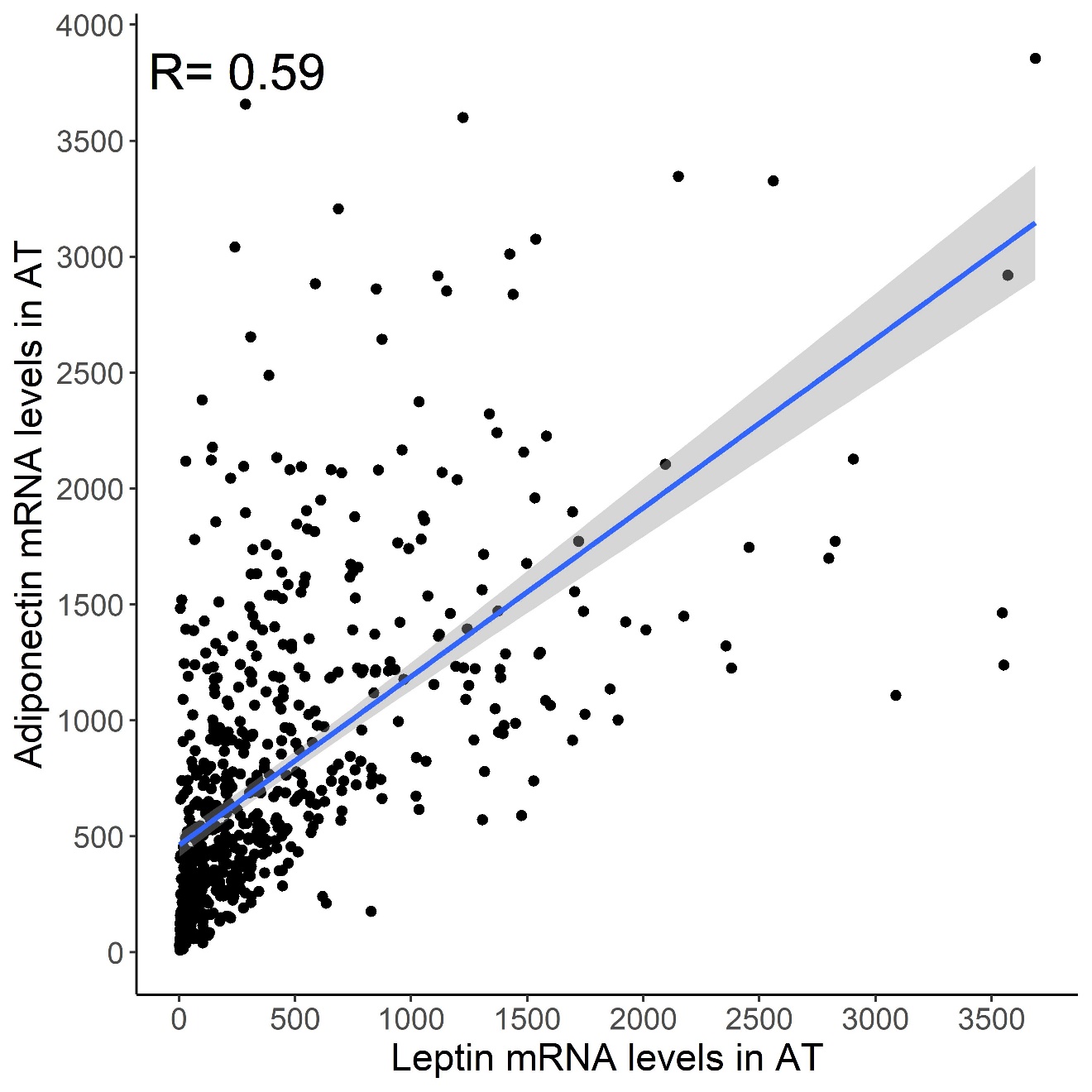


Supplementary Figure 5. Correlation between adiponectin and leptin mRNA levels in subcutaneous adipose tissue based on publicly available data for 661 individuals from the GTEx Portal (<https://gtexportal.org/home/>).
